# Longitudinal evaluation of hemodynamic blood and echocardiographic biomarkers for the prediction of BPD and BPD-related pulmonary hypertension in very-low-birth-weight preterm infants

**DOI:** 10.1007/s00431-024-05841-8

**Published:** 2024-11-15

**Authors:** Lukas Schroeder, Fabian Ebach, Tamene Melaku, Brigitte Strizek, Jorge Jimenez-Cruz, Ramona Dolscheid-Pommerich, Andreas Mueller, Florian Kipfmueller

**Affiliations:** 1 Department of Neonatology and Pediatric Intensive Care Medicine, University Children’s Hospital Bonn, Venusberg-Campus 1, 53127 Bonn, Germany; 2 Department of Obstetrics and Prenatal Medicine, University Hospital Bonn, Bonn, Germany; 3 Institute of Clinical Chemistry and Clinical Pharmacology, University Hospital Bonn, Bonn, Germany

**Keywords:** Bronchopulmonary dysplasia, Pulmonary hypertension, Preterm infants, Biomarker, Echocardiography, Mortality

## Abstract

Very-low-birth-weight infants (VLBW, < 1500 g) are at risk of developing bronchopulmonary dysplasia (BPD) and are at risk for BPD-related pulmonary hypertension (PH). The longitudinal measurement of innovative blood and echocardiographic biomarkers might allow for a risk stratification of these infants. A prospective single-center cohort study was conducted between 01/2021 and 06/2023. Inclusion criteria were the combination of a birth weight < 1500 g and a gestational age (GA) ≤ 30/0 weeks. Assessment timepoints: T1 (day 7), T2 (day 28), and T3 (at 36 weeks post-menstrual age, PMA). Overall, 71 preterm infants were included for final analysis. The *Z*log-transformed NTproBNP_Zlog_ (at T1 AUC 0.772; *p* = 0.019; at T2 AUC 0.874, *p* = 0.002), and endothelin-1 (ET1, at T1 AUC 0.789, *p* = 0.013) were identified as an early predictive biomarker for BPD/death in the univariate analysis. Additionally, echocardiographic markers of ventricular function and PH at T1 were predictive for BPD/death in the univariate analysis, with the highest predictivity found for the tricuspid annular plane systolic excursion-TAPSE (AUC 0.748, *p* = 0.016) and the pulmonary artery acceleration time to right ventricular ejection time (PAAT/RVET; AUC 0.744, *p* = 0.043). Regarding predictability of mortality alone NTroBNP_Zlog_ (at T1 AUC 0.973, *p* = 0.000), and CA125 (at T1 AUC 0.747, *p* = 0.008) were identified as potential predictors, as well as TAPSE (AUC 0.926, *p* = 0.000), and PAAT/RVET (AUC 0.985, *p* = 0.000) Several biomarkers including ET-1 (at T1 AUC 0.893, *p* = 0.000), TAPSE (AUC 0.974, *p* = 0.000), and PAAT/RVET (AUC 1.0, *p* = 0.000) at T1 were identified as univariate predictors for BPD-PH. In the multivariate analysis, no biomarker was identified as an independent predictor of the primary endpoint.

*Conclusion*: Mainly at an early stage of postnatal neonatal care in VLBW preterm infants, several biomarkers were found to be associated with the combined endpoint BPD/death and BPD-PH. New candidates of blood biomarkers (NTproBNPZ_log_, ET-1, and CA125) and echocardiographic markers (TAPSE, PAAT/RVET) might serve as innovative predictors for BPD, BPD-PH, and adverse outcomes in VBLW infants.

**Table:** 

**What is Known:**	
• *VLBW infants are at risk for the development of BPD and BPD-related PH, which both are main contributors for short and long-term morbidity and mortality. Several studies in the past focused on the evaluation of circulating blood biomarkers and biomarkers from echocardiographic assessment of these infants. But to date, there is still a lack on longitudinal prospective studies especially in VLBW infants.*	
**What is New:**	
• *For the first time, this set of selected blood biomarkers (with the first description of Zlog-transformed NTproBNP and CA125 in preterm infants) and several echocardiographic markers were analyzed in a prospective longitudinal study from birth until 36 weeks post menstrual age in VLBW infants. Our data help clinicians to identify preterm infants at risk for BPD, BPD-PH and death and to offer new candidates of biomarkers. This might help to facilitate decision making and guidance of therapy in these highly vulnerable patients.*

**Supplementary Information:**

The online version contains supplementary material available at 10.1007/s00431-024-05841-8.

## Introduction

Very-low-birth-weight infants (< 1500 g, VLBW) and especially extremely low gestational age newborns (< 28 weeks of gestational age, ELGAN) are at risk of developing bronchopulmonary dysplasia (BPD), which is the most frequent chronic lung disease in preterm infants [1, 2]. The reported incidences among ELGAN and VLBW infants in the USA are 36.9% (BPD grades 1–2) and 3.7% (BPD grade 3) in preterm infants with a gestational age (GA) of 22/0–28/6 weeks [2]. In a meta-analysis of the global incidences of BPD, the authors reported a wide range of BPD incidences (17–73%) for European countries, with an even wider range of 18–89% for North America [3]. Despite the efforts of the research community over the last decades to prevent BPD and to optimize treatment strategies for BPD, the global BPD incidences and mortality of infants suffering from BPD remain high [4].

In recent years, multiple studies were conducted analyzing predictors for the early identification of BPD in preterm infants. Two possible approaches for the prediction of BPD are (I) circulating serum and plasma biomarkers and (II) echocardiographic biomarkers of pulmonary hypertension (PH) and right and left ventricular dysfunction (diastolic and systolic RVD/LVD). PH related to BPD (BPD-PH) is one of the major contributors to short and long-term morbidity and mortality in preterm infants, as about 25–30% of infants with moderate to severe BPD develop BPD-PH [5, 6]. Besides, diastolic and systolic RVD and LVD are known pathologies seen in retrospective and prospective cohorts of preterm infants with BPD or BPD-PH [7, 8], as measured by echocardiographic techniques such as pulsed-wave (PW) Doppler, M-mode, tissue Doppler imaging (TDI), or speckle tracking. Multiple serum and plasma biomarkers have already been proven as robust predictors for BPD/death or BPD-PH in preterm infants (e.g., n-terminal pro b-type natriuretic peptide [NTproBNP], endothelin-1 [ET-1], intercellular adhesion molecule 1 [ICAM-1], soluble fragment of cytokeratin 19 [Cyfra 21–1], and kallistin) [9–12]. However, the recent research data lack longitudinal prospective measurements (from day 7 up to 36 weeks post-menstrual age [PMA]) of circulating biomarkers in combination with echocardiographic biomarkers in cohorts of preterm infants. Additionally, to date, there are novel circulating biomarkers as the *Z*log-transformed NTproBNP_zlog_ and the carbohydrate antigen 125 (CA125) that until now have not been evaluated in preterm infants at risk for BPD. The major aim of this study was to focus on the predictivity of longitudinal blood and echocardiographic biomarker measurements at multiple timepoints and stages of disease evolution during neonatal intensive care treatment.

## Material and methods

### Patient information

VLBW preterm infants treated at the neonatal intensive care unit (NICU) of the University Children’s Hospital of Bonn, Germany, during the study period 09/2021–06/2023 were prospectively screened for study participation. Inclusion criteria were birth weight (BW) < 1500 g, ≤ 30/0 weeks of GA, signed informed parental consent, and at least one echocardiographic assessment in combination with a sample of circulating biomarkers at the defined timepoints as specified below. Patients were excluded from the study, if they met at least one of the following criteria: ≥ 30/1 weeks of GA and BW > 1500 g, early death (< 7 days of life), primary palliative care, or congenital heart defects (CHD) requiring surgical repair. A patent ductus arteriosus (PDA), an atrial septal defect (ASD, < 5 mm), and a muscular ventricular septal defect (VSD, < 2 mm) were not classified as relevant CHD.

## Patient consent and ethical approval

The study was approved by the local ethics committee of the Medical Center of the University of Bonn (local running number 011/19). Patients were prospectively enrolled in the study after informed written consent was obtained from the parents or legal representative. The methods used for the clinical research were performed in accordance with the STROBE (strengthening the reporting of observational studies in epidemiology) guidelines [13] and in accordance with the Declaration of Helsinki. The study was registered at the German Clinical Trials Register (GCT; trial number, DRKS00033219).

### Diagnostic assessment

#### Timepoints of blood samples and biomarker measurements

Blood samples (serum and plasma which was centrifugated from EDTA blood) were taken at the following timepoints: (1) T1 (day of life [DOL] 7, + / − 1 day), (2) T2 (DOL 28, + / − 2 days), (3) T3 (at 36 weeks PMA, + / − 2 days). Blood samples were collected when routine blood sampling was performed as indicated by the attending physician. Blood samples were collected via an indwelling arterial line catheter (Art. radialis or Art. tibialis posterior) or via peripheral venous puncture. The specific description of biomarker measurements and analyzing techniques for NTproBNP_Zlog_, Cyfra 21–1, ET-1, and carbohydrate antigen 125 (CA125) is described in the online supplemental material (*Description of hemodynamic biomarkers and definitions*).

#### Echocardiographic assessment and targeted parameters

For all echocardiographic measurements, a Philips CX50 Compact Extreme Ultrasound system with a S12-4 sector array transducer (Philips Healthcare, Best, the Netherlands) was used. After informed written consent and patient recruitment, VLBW infants received a standardized neonatal echocardiographic examination at T1, T2, and T3. To minimize interobserver variation, all echocardiographic examinations within this study were performed by one of two senior neonatologists highly experienced in neonatal echocardiography. Images and cine loops were stored and reviewed offline for the calculation of echocardiographic indices. Echocardiographic markers of PH as well as markers of ventricular dysfunction were assessed according to recently published guidelines for targeted neonatal echocardiography and were described previously in detail by our study group [14–17]. The assessment and definition of these echocardiographic markers are explained in detail in the online supplemental material (*Description of hemodynamic biomarkers and definitions*).

#### Outcome measures and sample size estimation

The combined outcome of BPD/ death was defined as the combined primary endpoint. BPD was defined as oxygen dependence for 28 days and respiratory support at 36 weeks PMA according to the definition of Jobe and Bancalari [18]. Accordingly, infants were allocated to group A (BPD/death) and to group B (no BPD/no death). The following parameters were defined as secondary outcome measures: BPD alone, in-hospital mortality (death), and BPD-PH (36 weeks of PMA). As no comparable data sets for the multiple and longitudinal measurements of these biomarker combinations were available, we did not perform a power calculation. We aimed to reach a sample size of 76 infants (with an allocation ratio of 5:1 for no BPD/BPD, group 1 63 vs group B 13), with a moderate to a high effect size of 0.8, and an alpha level of 0.05 to reach a power of 80%.

### Statistical analysis

For the comparison of continuous and non-normally distributed variables, a Wilcoxon test or Mann–Whitney *U* test was performed to compare continuous variables between two timepoints and subgroups or a general linear model with an ANOVA for repeated measurements (with Bonferroni Holm correction). For categorical variables, the Pearson’s chi^2^ test and Fisher’s exact test were applied, as appropriate. For correlation analysis, normally distributed data were analyzed using Pearson’s correlation analysis, and if non-normally distributed, using Spearman correlation analysis between continuous variables. Receiver-operating-characteristic (ROC) analysis was used for the prediction of the primary endpoint and the Kolmogorov–Smirnov test for the optimal cutoff value and identification of the best combination between sensitivity and specificity. A binary regression model was used to adjust blood biomarkers and echocardiographic markers for covariables (GA, duration of MV, sepsis). Metric variables included in the model wer normalized with the natural logarithm (ln) using the Box-Tidwell method. A *p*-value of < 0.05 was considered significant. The statistical analysis was performed using statistical software (IBM SPSS Statistics for Windows, Version 27.0., IBM Corp, Armonk, NY).

## Results

Overall, 71 VLBW infants were included in the final analysis, as shown with the exact number of sample volumes at timepoints T1–T3 in the flow-chart in online supplemental Fig. 1. The epidemiological characteristics, feto-maternal diagnosis, and data on comorbidities are displayed in Table 1.

**Table 1:** Demographic and treatment data

Variables	Overall cohort *n* = 71	Group A (BPD/death) *n* = 18 (25)	Group B (no BPD/no death) *n* = 53 (75)	*p*-level
*Gestational age, w*	26.9 (2.2)	25 (2.4)	27.5 (1.7)	** < 0.001**
*Female sex, n (%)*	39 (54)	8 (44)	31 (59)	0.421
*Birth weight, kg*	0.87 (0.28)	0.61 (0.18)	0.96 (0.26)	** < 0.001**
*ELGANs, n (%)*	44 (62)	15 (83)	29 (55)	**0.048**
*ELBW infants, n (%)*	52 (73)	18 (100)	34 64)	**0.002**
APGAR 5 min*	8 (7/9)	7 (5/9)	8 (5/10)	**0.002**
APGAR 10 min*	9 (9/9)	8 (3/10)	9 (6/10)	**0.002**
*Antenatal corticosteroid therapy, n (%)*	51 (71)	11 (61)	40 (76)	0.363
*Surfactant therapy 1 d, n (%)*	61 (85)	17 (94)	44 (83)	0.434
***Feto-maternal diagnosis***				
(a) *PPROM, n (%)* (b) *Preterm labor, n (%)* (c) *Preeclampsia, n (%)* (d) *IAI, n (%)* (e) *Fetal growth restriction, n (%)*	13 (18) 36 (51) 10 (14) 10 (14) 12 (17)	5 (27) 13 (72) 2 (11) 3 (17) 5 (27)	8 (15) 23 (43) 8 (15) 7 (13) 7 (13)	0.292 0.055 0.99 0.706 0.166
***Comorbidities and treatment data***				
*nRDS grade 3–4, n (%)*	12 (17)	9 (50)	3 (6)	**0.004**
*Intraventricular hemorrhage*	13 (18)	7 (39)	6 (11)	**0.015**
*Necrotizing enterocolitis*	4 (6)	1 (6)	3 (6)	0.99
*Retinopathy of prematurity, n (%)*	32 (46)	6 (35)	26 (49)	0.406
*Clinical sepsis, n (%)*	27 (38)	10 (56)	17 (32)	0.096
*Blood culture positive sepsis, n (%)*	10 (14)	7 (39)	3 (6)	**0.002**
*Invasive MV, n (%)*	23 (32)	11 (61)	12 (23)	**0.007**
*Duration of MV, d*	10.7 (13)	18 (17)	4.1 (2.6)	**0.019**
*Oxygen supplementation, d*	21.6 (34)	49 (44)	7 (10)	** < 0.001**
*iNO treatment, n (%)*	10 (14)	8 (44)	2 (4)	** < 0.001**
*BPD at 36 weeks PMA, n (%)* *(a) Mild* *(b) Moderate* *(c) Severe*	11 (17) 5 (8) 4 (6) 2 (3)	11 (85) 5 (39) 4 (31) 2 (15)	0	
*Vasoactive treatment, n (%)*	40 (56)	16 (88)	24 (45)	**0.002**
*PH at 36 weeks PMA, n (%)*	3 (7)	2 (11)	1 (2)	0.163
*In-hospital stay, d*	73 (37)	82 (54)	70 (29)	0.266
*In-hospital mortality, n (%)*	8 (11)	8 (44)	0	

Data are demonstrated as absolute number with percentage, as mean values with standard deviation (+ / −) (normally distributed data), or as median with IQR (25/75) (non-normally distributed). Parameters with a *p*-level < 0.05 are highlighted in bold. Clinical sepsis is defined as the requirement of an antibiotic therapy for at least 5 days and clinical signs of a bloodstream infection. Abbreviations: *IAI*, intra-amniotic infection; *BPD*, bronchopulmonary dysplasia; *DOL*, day of life; *ELGAN*, extremely low gestational age newborns (< 28 weeks of GA); *ELBW*, extremely low birth weight (< 1000 g); *MV*, mechanical ventilation; *iNO*, inhaled nitric oxide; *PMA*, post-menstrual age; *PPROM*, preterm premature rupture of membrane (> 24 h preterm to labor); *PH*, pulmonary hypertension; *nRDS*, neonatal respiratory distress syndrome. The asterisk is illustrating non-normally distributed data

### Longitudinal biomarker samples

**Fig. 1:**
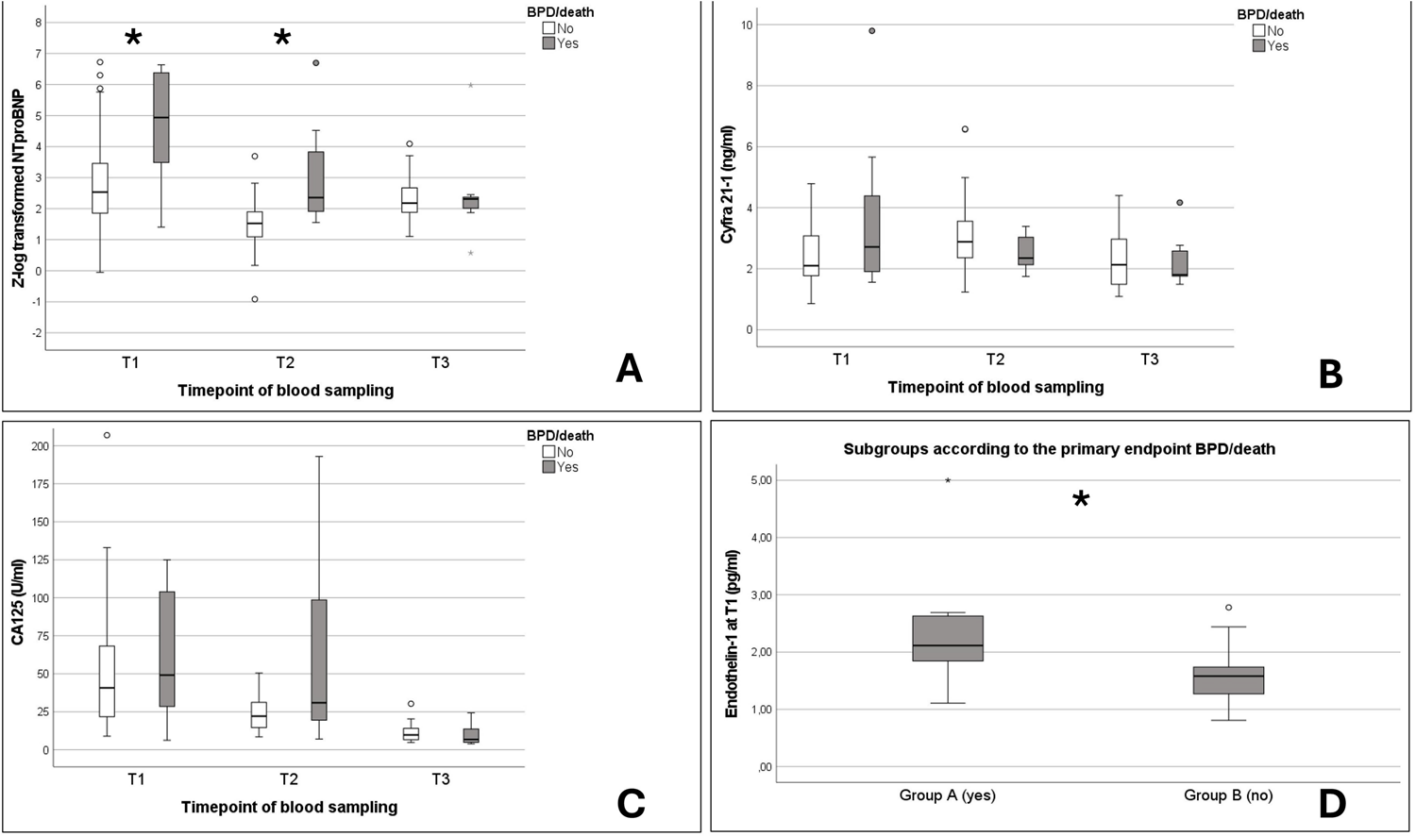
Longitudinal data on blood biomarker measurements are displayed as mean values in Figs. 2A–D, with the corresponding values for Group A (BPD/death) and Group B (no BPD/death). **A** *Z*log-transformed NTproBNP (correction for gestational age) values at T1–T3. **B** Cyfra 21–1 values at T1–T3. **C** CA125 values at T1–T3. **D** Endothelin-1 values at T1. Asterisks are indicating significant *p*-values (< 0.01) between subgroups

The mean values of the longitudinal biomarker measurements as well as the correlation analysis with the primary outcome parameter are displayed in Fig. 1 and online supplemental Table 1. In summary, infants experiencing the primary endpoint (group A) had significantly higher NTproBNP_Zlog_ levels at T1 and T2 compared to group B. Additionally, ET-1 levels differed significantly at T1 between subgroups, with higher values in group A. Only at T1 blood samples of ET-1 were available (*n* = 35; no-BPD = 23; BPD = 12). Cyfra 21–1 and CA125 values (T1–T3) did not differ significantly between groups. In the correlation analysis, NTproBNP_Zlog_ as well as ET-1 correlated significantly with echocardiographic markers of RV-, LV-dysfunction, and RV-pulmonary artery (PA) coupling (see Table 2). In the multivariate analysis, NTproBNP_Zlog_ failed to reach significance as an independent predictor of the combined endpoint (see Table 3).

**Table 2:** Biomarker correlation analysis

Variables	*N*	TAPSE at T1 *r (p-level)*	*E′-Wave LV at T1* *r (p-level)*	*E′/A′-Wave LV at T1* *r (p-level)*	PAAT/RVET at T1 *r (p-level)*
*NT-proBNP*_*Zlog*_ at T1	*50*	** − 0.430 (0.014)**	** − 0.325 (0.034)**	− 0.250 (0.106)	** − 0.486 (< 0.001)**
Cyfra 21–1 at T1*	*60*	− 0.306 (0.083)	− 0.025 (0.869)	− 0.096 (0.527)	− 0.025 (0.857)
CA125 at T1*	*60*	− 0.129 (0.473)	− 0.052 (0.733)	− 0.099 (0.519)	− 0.094 (0.517)
Endothelin-1 at T1*	*35*	− 0.207 (0.383)	− 0.321 (0.090)	− 0.021 (0.931)	** − 0.385 (0.030)**

Spearman correlation (between non-normally distributed data) and Pearson correlation (between normally distributed data) were performed for the calculation of the correlation coefficient. *p*-levels < 0.05 are highlighted in bold. Abbreviations: *A′*, late diastolic wave indicating the active atrial contraction in the tissue Doppler imaging; *CA125*, carbohydrate antigen 125; *E′*, early diastolic wave indicating the passive ventricular filling in the tissue Doppler imaging; *N*, included samples per parameter; *NTproBNP*_*Zlog*_, *Z*log-transformed N-terminal pro-brain natriuretic peptide; *PAAT*, pulmonary artery acceleration time; *RVET*, right ventricular ejection time; *TAPSE*, tricuspid annular plane systolic excursion; *T1*, day of life 7). The asterisk is illustrating non-normally distributed data

**Table 3:** Binary logistic regression model

*Regression model quality* *BPD/death*	*Model 1* *OR 17.1, p* = *0.009, Cox R*^*2*^*0.35*	
	***OR (95%CI)***	***p-level***
Gestational age	0.014 (0/22680)	0.561
*Duration of MV*	1.3 (0.7/2.4)	0.387
*Sepsis*	1.3 (0.12/13)	0.850
*NTproBNP*_*Zlog*_* at T1*	0.6 (0.04/8.1)	0.662
*PAAT/RVET at T1*	0.019 (0/1.2)	0.064
*E′/A′LV (TDI)*	0.625 (0.11/37)	0.822

A binary logistic regression model was performed with simultaneous inclusion of all covariates. Metric variables were transformed with the natural logarithm (ln) using the Box-Tidwell method. Parallel inclusion of more significant biomarkers or echocardiographic markers led to higher rates (correlation coefficient > 0.7) of multi-collinearity, and other parameters were excluded. Variables with a *p*-level < 0.01 in the univariate analysis were included. Endothelin-1 values were not included due to lower numbers of blood samples. *p*-levels = or < 0.05 are highlighted in bold. Abbreviations: *MV*, mechanical ventilation; *NTproBNP*_*Zlog*_, *Z*log-transformation of N-terminal pro-brain natriuretic peptide; *PAAT*, pulmonary artery acceleration time; *RVET*, right ventricular ejection time; *A′*, late diastolic wave indicating the active atrial contraction in the tissue Doppler imaging; *E′*, early diastolic wave indicating the passive filling of the ventricular filling in the tissue Doppler imaging; *LV*, left ventricle

### ROC-analysis of longitudinal biomarker samples

**Fig. 2:**
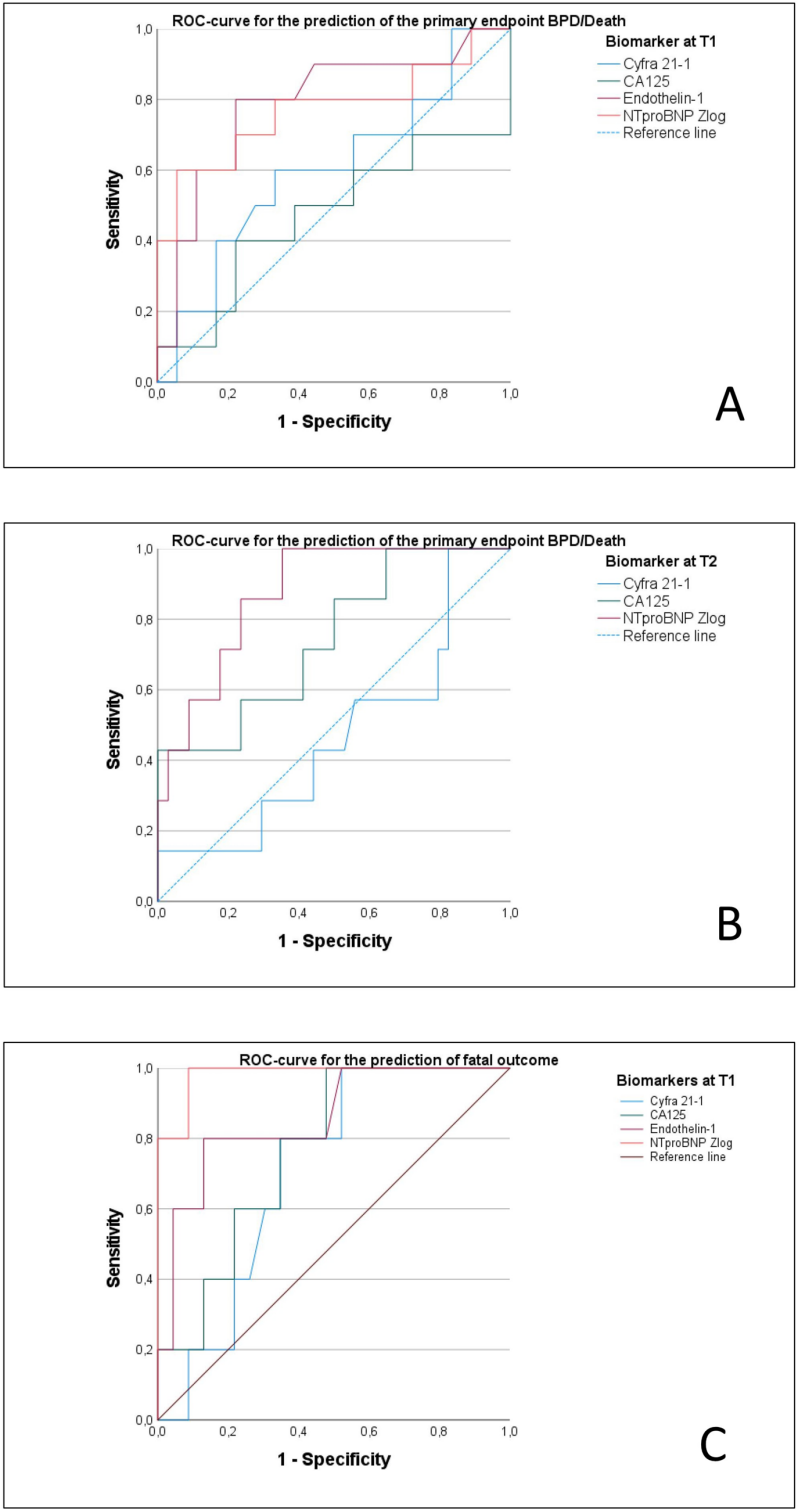
Illustration of the receiver operating characteristics (ROC) curves for the prediction of the primary combined endpoint BPD/death (**A** T1; **B** T2) and fatal outcome separately (**C** T1) using blood biomarkers

The ROC-curve analysis is illustrated in Fig. 2A–C. Only biomarkers with a *p*-level < 0.01 were included in the final ROC analysis. At T1, the ROC analysis revealed NTproBNP_Zlog_ (AUC 0.772; *p* = 0.019), and ET-1 (AUC 0.789, *p* = 0.013) as an appropriate biomarker to predict the primary endpoint. The optimal cutoffs at T1 were calculated as follows: NTproBNP_Zlog_ 2.6 (sensitivity 60%, specificity 94%) and ET-1 1.8 pg/ml (sensitivity 80%, specificity 78%).

At T2, NTproBNP_Zlog_ (AUC 0.874, *p* = 0.002) was identified as robust predictors for the primary endpoint. The optimal cutoff value for NTproBNP_Zlog_ was 0.43 (sensitivity 100%, specificity 65%). At T3, no biomarker reached statistical significance as a predictor for the primary endpoint.

The results of the subgroup analysis regarding the outcome parameter death alone at T1 are illustrated in Fig. 2C. The parameter NTproBNP_Zlog_ (AUC 0.973, *p* = 0.000) and CA125 (AUC 0.747, *p* = 0.008) were identified as significant predictors of fatal outcome, but not for BPD alone (including Cyfra 21–1 and ET-1). When looking for predictors for the development of BPD-PH (diagnosis of PH at 36 weeks PMA) ET-1 at T1 was identified as a predictor for BPD-PH (AUC 0.893, *p* = 0.000) (see online supplemental Fig. 2B).

### Longitudinal echocardiographic assessment

**Fig. 3:**
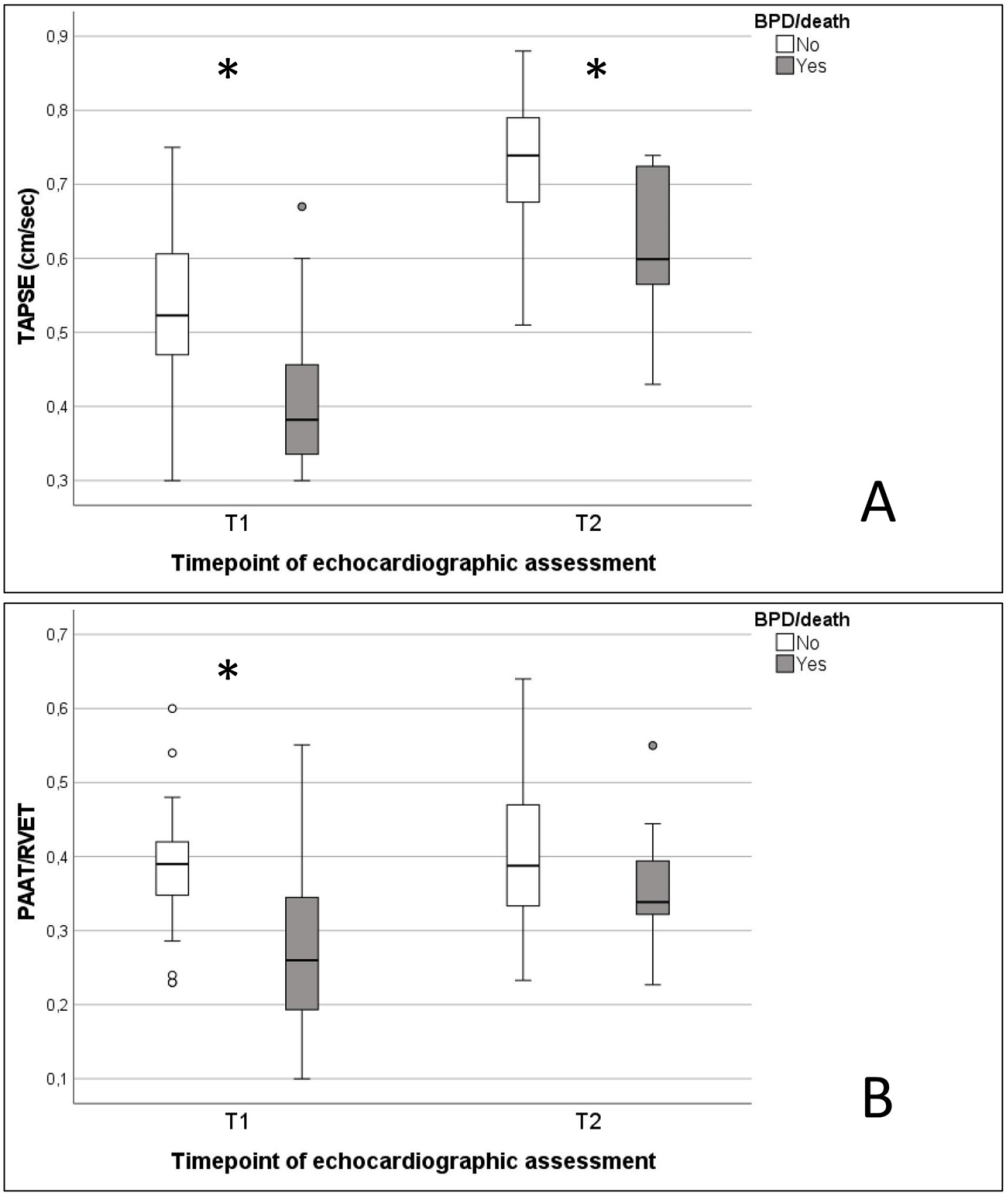
**A** Illustration of the mean values of TAPSE (tricuspid annular plane systolic excursion, cm/sec) at T1 (day 7) and T2 (day 28) with the corresponding values for Group A (BPD/death) and Group B (no BPD/death) and **B** mean values of PAAT/RVET (pulmonary artery acceleration time/right ventricular ejection time) at T1 and T2. Asterisks are indicating significant values (*p *<0.05) between subgroups

The mean values of all echocardiographic parameters in the overall cohort and for the respective subgroups are displayed in the online supplemental Table 2. Data on RV systolic function (TAPSE) and pulmonary artery flow characteristics (PAAT/RVET) are illustrated separately in Fig. 3A, B. At T1, the incidences of PH (28% vs 4%, *p* = 0.010) and RVD (28% vs. 4%, *p* = 0.010) differed significantly between subgroups, with higher incidences in infants allocated to group A. Both at T2 and T3 (BPD-PH), the incidence of PH was higher in group A (11% vs 2% at both T2 and T3), without statistical significance. LVD and BVD did not differ significantly between groups A and B (T1–T3). Furthermore, the incidence of a patent ductus arteriosus (PDA) and the RV/LV ratio did not differ between groups A and B at all timepoints (T1–T3).

At T1 TAPSE, markers of diastolic ventricular function, and pulmonary artery flow characteristics (PAAT, PAAT/RVET) differed significantly between subgroups (see online supplemental Table 2). At T2 TAPSE, makers of systolic as well as diastolic ventricular function were significantly lower in group A. At T3, no significant differences were found between subgroups. In the multivariate analysis, PAAT/RVET and E′/A′LV (TDI) failed to reach significance as independent predictors of the combined endpoint BPD/death (see Table 3).

### ROC analysis for longitudinal echocardiographic parameters

The ROC-curve analysis at T1 is illustrated in online supplemental Fig. 2A. Only echocardiographic parameters with a *p*-level < 0.01 were included in the final ROC analysis. The ROC analysis at T1 revealed the following AUC values as predictors for the primary endpoint: TAPSE (AUC 0.748, *p* = 0.016), E′/A′ wave LV (TDI, AUC 0.744, *p* = 0.014), PAAT (AUC 0.740, *p* = 0.047), and PAAT/RVET (AUC 0.744, *p* = 0.043).

The optimal cutoffs were calculated as follows: TAPSE 0.45 cm (sensitivity 85%, specificity 70%), E′/A′ wave LV (TDI) 0.66 (sensitivity 89%, specificity 60%), PAAT 48.5 ms (sensitivity 96%, specificity 60%), and PAAT/RVET 0.36 (sensitivity 73%, specificity 80%).

The results of the subgroup analysis regarding BPD and death at T1 as separate outcome parameters are as follows: TAPSE (AUC 0.926, *p* = 0.000), E′/A′ wave LV (AUC 0.853, *p* = 0.000), PAAT (AUC 0.971, *p* = 0.000), and PAAT/RVET (AUC 0.985, *p* = 0.000) were identified as significant predictors of fatal outcome. Additionally, the parameter E′/A′ wave LV (AUC 0.771, *p* = 0.02) was found to be predictive for BPD at 36 weeks PMA. When further subclassifying for predictors of BPD-PH, TAPSE (AUC 0.974, *p* = 0.000), E′/A′ wave LV (AUC 0.798, *p* = 0.002), PAAT (AUC 1.0, *p* = 0.000), and PAAT/RVET (AUC 1.0, *p* = 0.000) at T1 were identified as predictors for BPD-PH (see online supplemental Fig. 2C).

## Discussion

The key findings of the present longitudinal observational study can be summarized as follows: NTproBNP_Zlog_ (T1 + T2) and ET-1 (T1) were identified as early predictive biomarkers (DOL 7 and DOL 28) for the combined endpoint BPD/death in VLBW-infants in the univariate analysis. In a subgroup analysis, NTproBNP_Zlog_ and CA125 at T1 (DOL 7) could be identified as predictors for in-hospital mortality, but not for BPD alone. ET-1 at T1 was predictive for the development of BPD-PH at 36 weeks of PMA, as well as the following echocardiographic parameters at T1: TAPSE, E′/A′ wave LV, PAAT, and PAAT/RVET. Regarding the echocardiographic assessment, several markers could be identified as significant predictors for the combined primary endpoint at T1, T2, and T3 in the univariate analysis. After adjusting for GA, duration of MV, and neonatal sepsis, no biomarker was identified as an independent predictor of the primary endpoint.

### Blood biomarkers findings in critical review of the literature

NTproBNP was already shown to correlate with the outcome parameter BPD/death or BPD alone in multiple studies [19–24]. The timepoint of NTproBNP measurement differed among these studies, analyzing plasma samples at DOL 1 to 36 weeks PMA. Most of these studies focused on VLBW infants < 30–32 weeks of GA. Recently, NTproBNP samples were analyzed in a longitudinal observational study on VLBW infants at DOL 1, 3, 7, and later on weekly until DOL 49 after birth [20]. The authors concluded that NTproBNP levels obtained at DOL 14–35 are predictive for the development of BPD, even when adjusting for the presence of a hemodynamic-relevant PDA. According to our findings, NTproBNP_Zlog_ levels in the first week of life (DOL 7) did not differ when subclassifying for BPD alone. However, when looking for the combined endpoint BPD/death and for death alone, NTproBNP_Zlog_ was identified as a univariate predictor at DOL 7. Other studies revealed concordant data and identified NTproBNP as a predictor for BPD and/or death in the first weeks of life [25, 26]. Additionally, NTproBNP was identified as an early predictor for the development of a BPD-PH in preterm infants [27]. In our cohort, NTproBNP_Zlog_ (T1–T3) was not predictive for the development of BPD-PH, whereas ET-1 at T1 was identified as a predictor for BPD-PH. For the first time, we described the *Z*log-transformed NTproBNP as an early biomarker for BPD/death in VLBW infants. The *Z*log-transformation of NTproBNP allows for age- and GA-independent interpretation of NTproBNP values and facilitates comparability and illustration of study data [28, 29]. To date, *Z*log-transformation of NTproBNP is not widely used, but in a previous study on preterm and term infants with PH and ventricular dysfunction, we could demonstrate the usefulness of NTproBNP_Zlog_ as new interpretation and illustration of classical NTproBNP values [30].

CA125 measured at T2 was found to correlate significantly with in-hospital mortality in VLBW infants but failed to predict the primary endpoint BPD/death or BPD-PH. Recently, CA125 was shown to be a robust predictor for in-hospital mortality in infants with a congenital diaphragmatic hernia (CDH) [31]. CA125 levels are known to decrease with GA due to decreasing expression in fetal tissues and increasing hepatic clearance during gestation, complicating the interpretation of CA125 in preterm infants. Besides GA, the volume status, pericardial and pleural effusions, and intestinal lesions/operations (NEC, intestinal perforation, or meconium ileus) might have an influence on CA125 values, and this might add bias on the interpretation of CA125 values in VLBW infants. Altogether, CA125 is an interesting cardiac biomarker, which has gained influence in the monitoring of acute and chronic heart failure as well as monitoring of PH in adult populations [32, 33].

ET-1 was shown to correlate in univariate analysis with the combined outcome parameter BPD/death in a large prospective two-center study with *n* = 231 patients [10] but failed as an independent predictor in multivariate analysis. A recent two-center prospective study came to the same conclusion, but both studies used different ELISA assays for ET-1 measurements, which makes interpretation of the results challenging [34]. However, these findings are in line with our findings, as we identified ET-1 as a potential predictor at T1 (DOL 7), with an appropriate AUC of 0.789 in the ROC analysis. Furthermore, the early measurement of ET-1 might serve as a predictor for BPD-PH in the present cohort.

As shown recently by our study group, Cyfra 21–1 serves as a predictor of BPD/death and prolonged MV in preterm infants [11]. According to the present data, we found a non-significant trend of higher Cyfra 21–1 levels in VLBW infants experiencing the BPD/death group. However, we could reproduce that preterm infants with prolonged MV expressed significantly higher Cyfra 21–1 levels than those with a shorter duration. The lower number of included infants, a differing timepoint of blood sampling, and the inclusion of preterm infants with older GA might explain differences in findings related to the primary endpoint between both studies.

### Echocardiographic biomarker findings in critical review of the literature

Several studies were conducted in recent years analyzing echocardiographic parameters as biomarkers for the combined endpoint of BPD/death and BPD-PH. An important fact influencing all studies on preterm infants with differing GA and differing BW is the ongoing maturation of hemodynamics, cardiac growth, and evolution of plateau values of echocardiographic markers displaying the diastolic and systolic function [21, 35]. Several of the before-mentioned echocardiographic parameters were already identified to correlate significantly with the evolution of BPD and fatal outcomes [36]. TAPSE is a reliable parameter with good interobserver robustness. The assessment of TAPSE is easy to learn and was identified as an independent predictor of BPD/death or BPD-PH in VBLW infants [21, 34, 37]. These data are supported by our longitudinal observations, revealing TAPSE to be significantly correlated with BPD/death and BPD-PH.

VLBW at risk for BPD are prone to a rapid evolution of RV or LV diastolic dysfunction, as compensatory mechanisms are limited due to high heart frequencies in comparison to term newborns, infants, and toddlers and lower contractile forces of atrial contraction [38]. When elevated LV preload (e.g., PDA) or increased LV afterload coincides with LV diastolic dysfunction in VLBW, this will potentially aggravate postcapillary pulmonary pressures and increase RV afterload. The interdependence between RV and LV as “dancing partners” illustrates the necessity for a broad assessment of diastolic and systolic ventricular dysfunction [39]. TDI measurements allow for a sensitive assessment of myocardial dysfunction in preterm infants. Recent studies revealed an association of TDI-related measures (E′ wave, A′ wave, E′/A′ wave, or E/E′ ratio) and BPD/death in VLBW infants [7, 21]. Nevertheless, there is still a lack on longitudinal data of TDI-imaging in VLBW infants. Our research adds new data on longitudinal TDI measurements, with the identification of an impaired diastolic as well as systolic (S wave) ventricular function (RV and LV) in VLBW infants experiencing BPD/death and BPD-PH, both at an early stage (T1) and later stages of in-hospital stay (T2 and T3).

The efficacy of the RV function depends on the function of the pulmonary vascular (PV) compliance, and this interaction is known as RV-PV coupling, as described elsewhere and with growing interest in the research community [39]. There is a lack of analysis in cohorts of preterm infants and the study conducted by Bussmann et al. gave insights into the efficacy of the non-invasive assessment using echocardiographic makers to assess the RV length–force relationship with pulmonary artery flow characteristics (TAPSE/PAAT). Pulmonary artery flow characteristics allow for the estimation of elevated pulmonary vascular resistance (PVR) and identification of RV-PV coupling interference as well as PH estimation (systolic pulmonary artery pressure, sPAP) [39, 40]. Unexpectedly, we found no difference in RV-PV coupling between subgroups. This is interesting, as LV diastolic dysfunction was more apparent in infants experiencing BPD/death or BPD-PH and LV diastolic dysfunction is a known contributor to RV-PV uncoupling. The PAAT or PAAT/RVET depends on the presence of a PDA, but the assessment has a high intra- and interobserver agreement and high reliability, as shown in a recent multicenter study [40]. Our data are supportive that PAAT/RVET might be an early predictor for BPD/death and BPD-PH. At later stages (T2 + T3), PAAT/RVET failed to subclassify infants with BPD/death in our cohort.

The RV/LV ratio as an echocardiographic parameter for ventricular disproportion (RV-LV interaction) is easy to assess, and different approaches of assessment (in short-axis view or 4-chamber view) are described [36, 41]. In the present cohort, the RV/LV ratio did not differ between subgroups, although we expected significantly higher values in infants experiencing the primary endpoint. Nevertheless, we think that parameters such as the RV/LV ratio are easy to calculate in a targeted neonatal echocardiography and should be routinely used as screening parameters for RV-LV uncoupling in preterm and term infants.

The predictivity of blood and echocardiographic biomarkers varies when targeting different outcome parameters. Interestingly, the predictivity of early echocardiographic markers such as TAPSE or PAAT/RVET for BPD-PH is higher than for the primary outcome alone, as cardiac function might be impacted very early in preterm infants experiencing BPD-PH. These findings are in line with previous descriptions of early diagnostic approaches [42]. Similar findings were described for other imaging biomarkers like the early use (day 7 and day 14) of the lung-ultrasound score (LUS) as a predictor of BPD with a good diagnostic accuracy in predicting BPD (AUC 0.85–0.87) [43].

## Limitations

This study has several limitations, which need to be depicted. First, drop-outs (missing data) in the assessment of blood biomarkers have negatively impacted the data interpretation. The size of the subgroups related to the primary endpoint BPD/death and BPD-PH differed markedly. Only 10 infants in group A experienced the outcome of BPD at 36 weeks of PMA and 8 infants in group A died. Therefore, our data needs to be interpreted carefully. In the present study, preterm infants with BPD were identified using the older Jobe definition, according to the in-house standards. The actual Jensen definition (regardless of oxygen use) might discriminate better between BPD and no-BPD infants and therefore BPD rates might be underestimated. A multi-centric study with a predefined study set of identified biomarkers should be the next step to increase data robustness. The missing identification of independent predictors in the binary logistic regression model might be explained by the low inclusion rate and size of group A. The values of blood biomarkers depend strongly on post-processing after blood sampling from the patient. As NTproBNP, Cyfra 21–1, and CA125 are listed as routine parameters in the catalogue in our Institute of Clinical Chemistry, the processing of blood samples is without relevant time delay. Echocardiographic assessments strongly depend on inter- and intraobserver variation. This might have negatively influenced the analysis of our data set. To minimize this bias, only two senior neonatal echocardiographers were allowed to perform the echocardiographic assessments.

## Conclusion

Several blood biomarkers are associated with the primary endpoint BPD/death at an early (day 7 — NTproBNP_Zlog_, ET-1) and later stage (day 28 — NTproBNP_Zlog_) of postnatal management. ET-1 measured in the first week of life might be useful as a predictor for the later development of a BPD-PH. NTproBNP_Zlog_, ET-1, CA125, and Cyfra 21–1 as new biomarkers in VLBW infants need more attention in future biomarker studies for clarification of their significance. The assessment of diastolic and systolic ventricular function is essential in VLBW infants at different stages of the postnatal course, as makers such as TAPSE, PAAT/RVET, and TDI-derived markers allow for prediction of BPD, BPD-PH, and death in the VLBW cohort.

## Supplementary Information

Below is the link to the electronic supplementary material.

Supplementary file1 (DOCX 61 KB)

Supplementary file2 (DOCX 33 KB)

Supplementary file3 (DOCX 49 KB)

Supplementary file4 (JPG 407 KB)

Supplementary file5 (JPG 469 KB)

## Data Availability

The data that support the findings of this study are available on request from the corresponding author. The data are not publicly available due to privacy or ethical restrictions.
