# Supplementary material for: Longitudinal evaluation of hemodynamic blood and echocardiographic biomarkers for the prediction of BPD and BPD-related pulmonary hypertension in very-low-birth-weight preterm infants
Source: Eur J Pediatr. 2024 Nov 15;184(1):15. doi: 10.1007/s00431-024-05841-8 (PMC11567987; doi:10.1007/s00431-024-05841-8)
Supplement: Supplementary file 1 — Supplementary file1 (DOCX 61 KB) [file 431_2024_5841_MOESM1_ESM.docx]

# Description of Blood Biomarker and Analyzing Techniques

For one blood sample the total volume was ~0.8 ml (0.2ml EDTA for NTproBNP, 0.3ml for serum analysis of CA125, and Cyfra 21-1, 0.3ml plasma from centrifugated EDTA for Endothelin-1 analysis).

### NTproBNP measurements

NTproBNP plasma levels were measured using the electrochemiluminescence immunoassay (ECLIA) Elecsys proBNP II on a cobas e801 analyzer (Roche Diagnostics, Mannheim, Germany). In addition to the measurement of absolute values (pg/ml) NTproBNP values were transformed into Zlog values (NTproBNP_Zlog_, see formula below) for an easier GA-independent interpretation, as described previously by our study group in infants with PH and ventricular-dysfunction [1]. The formula was first described and evaluated by Palm et al. and Hoffmann et al. [2, 3]:

NTproBNP_Zlog_=$\frac{log x+0.512 x log t-3.417}{1.489 + 0.014 x log t}x 3.92$ (x=NTproBNP in pg/ml, t= age in days calculated as PMA).

1. Cyfra 21-1

Cyfra 21-1 (ng/ml) stands for the cytokeratin 19 fragment and is an integrative and structural subunit protein of epithelial intermediate filaments, which are integral components of the cytoskeleton [4]. Cyfra 21-1 serum levels were measured using the ECLIA Elecsys Cyfra 21-1 on a cobas e801 analyzer (Roche Diagnostics, Mannheim, Germany).

1. Endothelin-1

Endothelin-1 (pg/ml) is a peptide hormone and is released predominantly by endothelial cells and acts as potent vasoconstrictive mediator. Endothelin-1 was measured in EDTA plasma samples with an enzyme-linked immunoassay-sorbent-kit (ELISA, R&D-System, Minneapolis, United States of America). Endothelin-1 was measured from deep-frozen/ stored plasma-samples (-80°C).

1. Carbohydrate Antigen 125 (CA125)

CA125 (U/ml) is a glycoprotein and is synthetized by epithelial serous cells and is a well-known tumor marker in the management of ovarian cancer [5]. CA125 is also known as new biomarker for the monitoring of acute and chronic heart-failure in adult populations [6]. CA125 serum levels were measured using the ECLIA Elecsys CA1 25 II on a cobas e801 analyzer (Roche Diagnostics, Mannheim, Germany).

# Definitions of Feto-Maternal Diagnosis and Comorbidities

1. Intra-amniotic infection (IAI): this was defined as early neonatal sepsis immediately after birth and a suspected intra-uterine infection with signs of maternal and fetal systemic inflammation (eg, maternal tachycardia, uterine tenderness, maternal leukocytosis, malodorous vaginal discharge or amniotic fluid, and fetal tachycardia) [7]
2. Fetal growth restriction is defined as small for gestational age (SGA) fetuses, defined as estimated fetal weight (EFW) or abdominal circumference below the 3rd percentile [8].
3. Respiratory distress syndrome grade 3 is defined as confluent alveolar shadowing in the chest X-ray and grade 4 as alveolar shadowing obscuring the cardiac border [9].
4. Intraventricular hemorrhage is defined as (grade I) subependymal hemorrhages, (grade II) ventricular hemorrhages that fill less than 50 % of the ventricular lumen, (grade III) ventricular hemorrhages that occupy more than 50 % of the ventricular lumen. Echogenic proliferations in the parenchyma (grade IV hemorrhage), which correspond to hemorrhagic infarction [10].
5. Retinopathy of prematurity (ROP) was defined according to international definitions and guidelines and graded as ROP stage 1-5 (1 – demarcation line, 2 – ridge, 3 – extraretinal neovascular proliferations, 4 – partial retinal detachment, 5 – total retinal detachment) [11].
6. Necrotizing enterocolitis was diagnosed and defined as intraoperative finding of necrotized parts of the small intestine after an appropriate clinical condition of the preterm infant [12].

# Echocardiographic Assessment of PH and Ventricular Dysfunction

1. Pulmonary hemodynamics: PH was assessed using the following echocardiographic parameters: a) flow pattern of the ductus arteriosus (DA), with left-to-right, bidirectional, or right-to-left shunt; b) intraventricular septum (IVS) position, as normal, flattened, or D-shaped; and c) tricuspid valve regurgitation (TVR). Furthermore, the pulmonary artery acceleration time (PAAT, msec) and the right ventricular ejection time (RVET, msec) were assessed in a parasternal short-axis or subcostal view, based on PW-doppler measurement at the level distal the pulmonary valve. Additionally, the end-diastolic right-ventricular to left-ventricular (RV/LV) ratio was calculated in a standard four chamber directly distal to the tricuspid and mitral annulus as a horizontal line from endocardium of the RV and LV free wall to endocardium of the interventricular septum. The RV/LV-ratio was recently by proven as robust parameter for adverse outcome in infants with congenital diaphragmatic hernia [13].
2. Measures of systolic and diastolic right, left, or biventricular dysfunction (RVD/LVD/BVD) were as follows: a) eyeball assessment in the apical four-chamber view; b) tricuspid annular plane systolic excursion (TAPSE) in the apical four-chamber view; c) PW Doppler measurement of the tricuspid and mitral valve inflow patterns for e and a wave calculation (cm/sec); d) TDI Doppler measurement of the lateral right, and left ventricular wall to calculate the S (systolic), E´, and A´ wave (diastolic); e) left ventricular ejection fraction (LVEF, %) according to the Teichholtz method in the parasternal long-axis view.

References

1. Schroeder L, Kuelshammer M, Dolscheid-Pommerich R, Holdenrieder S, Mueller A, Kipfmueller F (2023) NT-proBNP and Zlog-transformed NT-proBNP values predict extubation failure in critically ill neonates with pulmonary hypertension and ventricular dysfunction. Pediatric pulmonology 58(1):253–261. doi:10.1002/ppul.26193

2. Hoffmann G, Klawonn F, Lichtinghagen R, Orth M. (2017) The zlog value as a basis for the standardization of laboratory results. LaboratoriumsMedizin (41):23–32

3. Palm J, Hoffmann G, Klawonn F, Tutarel O, Palm H, Holdenrieder S, Ewert P (2020) Continuous, complete and comparable NT-proBNP reference ranges in healthy children. Clinical chemistry and laboratory medicine 58(9):1509–1516. doi:10.1515/cclm-2019-1185

4. Coulombe PA, Omary MB (2002) 'Hard' and 'soft' principles defining the structure, function and regulation of keratin intermediate filaments. Current opinion in cell biology 14(1):110–122. doi:10.1016/s0955-0674(01)00301-5

5. Canney PA, Moore M, Wilkinson PM, James RD (1984) Ovarian cancer antigen CA125: a prospective clinical assessment of its role as a tumour marker. British journal of cancer 50(6):765–769. doi:10.1038/bjc.1984.254

6. Soler M, Miñana G, Santas E, Núñez E, La Espriella R de, Valero E, Bodí V, Chorro FJ, Fernández-Cisnal A, D'Ascoli G, Marti-Cervera J, Sanchis J, Bayes-Genís A, Núñez J (2020) CA125 outperforms NT-proBNP in acute heart failure with severe tricuspid regurgitation. International journal of cardiology 308:54–59. doi:10.1016/j.ijcard.2020.03.027

7. Jung E, Romero R, Suksai M, Gotsch F, Chaemsaithong P, Erez O, Conde-Agudelo A, Gomez-Lopez N, Berry SM, Meyyazhagan A, Yoon BH (2024) Clinical chorioamnionitis at term: definition, pathogenesis, microbiology, diagnosis, and treatment. American journal of obstetrics and gynecology 230(3S):S807-S840. doi:10.1016/j.ajog.2023.02.002

8. Melamed N, Baschat A, Yinon Y, Athanasiadis A, Mecacci F, Figueras F, Berghella V, Nazareth A, Tahlak M, McIntyre HD, Da Silva Costa F, Kihara AB, Hadar E, McAuliffe F, Hanson M, Ma RC, Gooden R, Sheiner E, Kapur A, Divakar H, Ayres-de-Campos D, Hiersch L, Poon LC, Kingdom J, Romero R, Hod M (2021) FIGO (international Federation of Gynecology and obstetrics) initiative on fetal growth: best practice advice for screening, diagnosis, and management of fetal growth restriction. International journal of gynaecology and obstetrics: the official organ of the International Federation of Gynaecology and Obstetrics 152 Suppl 1(Suppl 1):3–57. doi:10.1002/ijgo.13522

9. Sefic Pasic I, Riera Soler L, Vazquez Mendez E, Castillo Salinas F (2023) Comparison between lung ultrasonography and chest X-ray in the evaluation of neonatal respiratory distress syndrome. Journal of ultrasound 26(2):435–448. doi:10.1007/s40477-022-00728-6

10. Deeg KH, Staudt F, Rohden L von (1999) Klassifikation der intrakraniellen Blutungen des Frühgeborenen (Classification of intracranial hemorrhage in premature infants). Ultraschall in der Medizin (Stuttgart, Germany : 1980) 20(4):165–170. doi:10.1055/s-1999-8898

11. Chiang MF, Quinn GE, Fielder AR, Ostmo SR, Paul Chan RV, Berrocal A, Binenbaum G, Blair M, Peter Campbell J, Capone A, Chen Y, Dai S, Ells A, Fleck BW, Good WV, Elizabeth Hartnett M, Holmstrom G, Kusaka S, Kychenthal A, Lepore D, Lorenz B, Martinez-Castellanos MA, Özdek Ş, Ademola-Popoola D, Reynolds JD, Shah PK, Shapiro M, Stahl A, Toth C, Vinekar A, Visser L, Wallace DK, Wu W-C, Zhao P, Zin A (2021) International Classification of Retinopathy of Prematurity, Third Edition. Ophthalmology 128(10):e51-e68. doi:10.1016/j.ophtha.2021.05.031

12. Patel RM, Ferguson J, McElroy SJ, Khashu M, Caplan MS (2020) Defining necrotizing enterocolitis: current difficulties and future opportunities. Pediatric research 88(Suppl 1):10–15. doi:10.1038/s41390-020-1074-4

13. Pugnaloni F, Bo B, Hale L, Capolupo I, Dotta A, Bagolan P, Schroeder L, Berg C, Geipel A, Mueller A, Patel N, Kipfmueller F (2023) Early Postnatal Ventricular Disproportion Predicts Outcome in Congenital Diaphragmatic Hernia. American journal of respiratory and critical care medicine 208(3):325–328. doi:10.1164/rccm.202212-2306LE
