# Supplementary material for: Longitudinal evaluation of hemodynamic blood and echocardiographic biomarkers for the prediction of BPD and BPD-related pulmonary hypertension in very-low-birth-weight preterm infants
Source: Eur J Pediatr. 2024 Nov 15;184(1):15. doi: 10.1007/s00431-024-05841-8 (PMC11567987; doi:10.1007/s00431-024-05841-8)
Supplement: Supplementary file 2 — Supplementary file2 (DOCX 33 KB) [file 431_2024_5841_MOESM2_ESM.docx]

Online supplemental Table 1: Longitudinal Biomarker Measurements

| **Variables** | **N** | **Overall cohort**  **n=71** | **Group A**  **(BPD/Death)**  **n=18 (25%)** | **Group B**  **(no BPD/no Death)**  **n=53 (75%)** | **p-level** |
| --- | --- | --- | --- | --- | --- |
| NT-proBNP_Zlog_   1. day 7 2. day 28* 3. 36 weeks PMA* | *50*  *43*  *36* | 3.3 (1.8)  1.6 (1.3/2.3)  2.4 (1.9/2.6) | 4.6 (1.9)  2.4 (1.9/4.5)  2.3 (1.9/2.4) | 2.8 (1.5)  1.5 (1.1/1.9)  2.2 (1.8/2.7) | **0.005**  **0.004**  0.886 |
| Cyfra 21-1   1. day 7* 2. day 28* 3. 36 weeks PMA* | *60*  *53*  *35* | 2.2 (1.8/3.6)  2.8 (2.2/3.4)  2.1 (1.5/2.8) | 2.7 (1.9/4.4)  2.4 (2.1/3.1)  1.8 (1.7/2.7) | 2.1 (1.8/3.1)  2.9 (2.3/3.6)  2.1 (1.5/3.0) | 0.114  0.449  0.810 |
| CA125   1. day 7* 2. day 28* 3. 36 weeks PMA* | *60*  *53*  *35* | 44 (21/96)  23 (15/35)  9 (6/14) | 49 (27/105)  31 (18/115)  7 (5/19) | 41 (22/75)  22 (15/31)  10 (7/14) | 0.448  0.117  0.323 |
| Endothelin-1   1. day 7* | *35* | 1.7 (1.4/2.1) | 2.1 (1.8/2.7) | 1.6 (1.2/1.8) | **0.002** |

Data are demonstrated as mean values with standard deviation (SD, +/-) (normally distributed data), or as median with IQR (25/75) (non-normally distributed). Parameters with a p-level <0.05 are highlighted in bold. Missing biomarker values occurred due to a) insufficient sample volume in EDTA-plasma or serum sample, b) critical hemodynamic status of the respective infant or neonatal anemia, with impossibility of blood sampling, or c) death/ discharge of the infant prior to blood sampling. Abbreviations: CA125: Carbohydrat Antigen 125; N: included samples per parameter, NTproBNP_Zlog_: Z-log transformed N-terminal pro-brain natriuretic peptide, PMA: post menstrual age)
