# Supplementary material for: Longitudinal evaluation of hemodynamic blood and echocardiographic biomarkers for the prediction of BPD and BPD-related pulmonary hypertension in very-low-birth-weight preterm infants
Source: Eur J Pediatr. 2024 Nov 15;184(1):15. doi: 10.1007/s00431-024-05841-8 (PMC11567987; doi:10.1007/s00431-024-05841-8)
Supplement: Supplementary file 3 — Supplementary file3 (DOCX 49 KB) [file 431_2024_5841_MOESM3_ESM.docx]

Online supplemental Table 2: Longitudinal Echocardiographic Assessment

| **Variables** | **N** | **Overall cohort**  **n=71** | **Group A**  **(BPD/Death)**  **n=18 (25%)** | **Group B**  **(no BPD/no Death)**  **n=53 (75%)** | **p-level** |
| --- | --- | --- | --- | --- | --- |
| PDA, n (%)   1. day 7 2. day 28 3. 36 weeks PMA | *71*  *54*  *42* | 10 (14)  5 (7)  3 (4) | 4 (22)  2 (11)  2 (11) | 6 (12)  3 (6)  1 (2) | 0.261  0.584  0.460 |
| RV/LV_enddiastolic_-Ratio, cm   1. day 7 2. day 28 3. 36 weeks PMA | *71*  *54*  *42* | 0.80 (0.17)  0.80 (0.12)  0.85 (0.15) | 0.91 (0.22)  0.81 (0.18)  0.89 (0.09) | 0.80 (0.15)  0.79 (0.09)  0.84 (0.16) | 0.076  0.714  0.245 |
| **Systolic RV/LV Function** | | | | | |
| TAPSE, cm   1. day 7* 2. day 28 3. 36 weeks PMA | *41*  *32*  *30* | 0.51 (0.39/0.60)  0.68 (0.11)  0.96 (0.14) | 0.38 (0.33/0.47)  0.62 (0.11)  0.99 (0.16) | 0.52 (0.46/0.61)  0.72 (0.09)  0.95 (0.13) | **0.006**  **0.034**  0.597 |
| LVEF, % (Teichholtz)   1. day 7 2. day 28 3. 36 weeks PMA | *37*  *22*  *31* | 73.9 (7.4)  71.3 (7.1)  69.6 (9.1) | 77.3 (4.1)  69.5 (8.8)  67.9 (9.9) | 72.9 (7.9)  72 (6.6)  70 (8.9) | 0.082  0.693  0.842 |
| **PW-Doppler Measurements** | | | | | |
| E/A-Wave TV   1. day 7 2. day 28 3. 36 weeks PMA | *61*  *32*  *31* | 0.69 (0.12)  0.68 (0.14)  0.79 (0.26) | 0.70 (0.13)  0.65 (0.07)  0.73 (0.12) | 0.69 (0.12)  0.69 (0.15)  0.81 (0.29) | 0.756  0.894  0.877 |
| E-Wave TV   1. day 7 2. day 28 3. 36 weeks PMA | *61*  *32*  *31* | 33.9 (6.5)  38.9 (9.1)  45.6 (10.8) | 34 (4.9)  40.3 (8.7)  44.9 (9.6) | 33.9 (7.0)  38.5 (10.0)  45.9 (11.4) | 0.819  0.656  0.877 |
| A-Wave TV   1. day 7 2. day 28 3. 36 weeks PMA | *61*  *32*  *31* | 50.4 (12.6)  58 (12.9)  60.5 (16.9) | 50.2 (10.6)  63.3 (10.7)  62.1 (14.8) | 50.5 (13.2)  56.8 (13.4)  59.9 (17.8) | 0.869  0.261  0.644 |
| E/A-Wave MV   1. day 7 2. day 28 3. 36 weeks PMA | *61*  *32*  *31* | 0.78 (0.18)  0.83 (0.19)  1.1 (0.39) | 0.69 (0.11)  0.67 (0.11)  0.97 (0.21) | 0.81 (0.20)  0.86 (0.19)  1.1 (0.44) | **0.049**  **0.017**  0.746 |
| E-Wave MV   1. day 7 2. day 28 3. 36 weeks PMA | *61*  *32*  *31* | 35.4 (10.1)  45.7 (10.3)  59 (14.9) | 31 (5.5)  36.8 (11.2)  56.1 (17.1) | 36.8 (10.9)  47.9 (9.1)  60.1 (14.2) | 0.079  **0.023**  0.672 |
| A-Wave MV   1. day 7 2. day 28 3. 36 weeks PMA | *61*  *32*  *31* | 46.1 (10.4)  56.9 (13.9)  59 (13.9) | 45 (8.1)  54.2 (9.2)  57.9 (14.2) | 46.5 (11.1)  57.6 (14.8)  59.4 (14.1) | 0.694  0.981  0.636 |
| **TDI-Measurements** | | | | | |
| E`/A`-Wave RV lateral   1. day 7 2. day 28 3. 36 weeks PMA | *60*  *34*  *36* | 0.62 (0.13)  0.75 (0.18)  0.92 (0.23) | 0.58 (0.11)  0.70 (0.22)  0.84 (0.28) | 0.64 (0.13)  0.76 (0.17)  0.95 (0.29) | 0.158  0.204  0.330 |
| E`-Wave RV lateral   1. day 7 2. day 28 3. 36 weeks PMA | *60*  *34*  *36* | 5.3 (1.6)  7.4 (1.9)  9.9 (2.3) | 4.6 (1.1)  7.8 (2.8)  8.9 (3.5) | 5.5 (1.6)  7.3 (1.5)  10.3 (1.7) | **0.035**  0.815  0.157 |
| A`-Wave RV lateral   1. day 7 2. day 28 3. 36 weeks PMA | *60*  *34*  *36* | 8.6 (2.2)  10.1 (2.2)  11.5 (3.9) | 8.1 (2.3)  11 (1.8)  10.8 (3.5) | 8.7 (2.2)  9.8 (2.4)  11.8 (4.0) | 0.308  0.135  0.596 |
| S-Wave RV lateral   1. day 7 2. day 28* 3. 36 weeks PMA | *60*  *34*  *36* | 5.6 (1.2)  7.0 (6.3/7.9)  8.2 (1.8) | 5.5 (1.2)  8.3 (6.5/9.2)  7.9 (2.3) | 5.9 (1.2)  6.9 (6.3/7.3)  8.3 (1.7) | 0.253  **0.046**  0.667 |
| E`/A`-Wave LV lateral   1. day 7 2. day 28 3. 36 weeks PMA | *60*  *34*  *36* | 0.85 (0.23)  0.85 (0.28)  1.1 (0.32) | 0.69 (0.18)  0.81 (0.25)  0.99 (0.41) | 0.89 (0.22)  0.86 (0.29)  1.08 (0.31) | **<0.001**  0.563  0.236 |
| E`-Wave LV lateral   1. day 7 2. day 28 3. 36 weeks PMA | *60*  *34*  *36* | 5.6 (1.8)  6.9 (2.7)  8.5 (2.1) | 4.3 (1.5)  7.0 (2.0)  7.4 (0.8) | 5.9 (1.7)  6.8 (1.7)  8.8 (2.4) | **0.002**  0.984  0.070 |
| A`-Wave LV lateral   1. day 7 2. day 28 3. 36 weeks PMA | *60*  *34*  *36* | 6.6 (1.6)  8.5 (2.3)  8.5 (2.3) | 6.5 (1.9)  8.6 (1.0)  8.5 (2.8) | 6.6. (1.5)  8.4 (2.5)  8.5 (2.3) | 0.765  0.460  0.864 |
| S-Wave LV lateral   1. day 7 2. day 28 3. 36 weeks PMA | *60*  *34*  *36* | 5.5 (1.2)  6.6 (1.2)  6.6 (1.6) | 5.3 (1.3)  7.3 (1.0)  7.0 (1.8) | 5.5 (1.2)  6.4 (1.2)  6.5 (1.5) | 0.586  0.064  0.837 |
| **Indices of RV/LV Diastolic Relaxation Impairment** | | | | | |
| E/E´-RV   1. day 7* 2. day 28 3. 36 weeks PMA | *60*  *34*  *36* | 6.6 (5.2/8.1)  5.2 (1.7)  4.9 (2.5) | 7.8 (6.4/9.5)  5.9 (1.2)  5.8 (2.5) | 6.2 (4.8/7.5)  5.1 (1.8)  4.5 (1.4) | **0.018**  0.143  **0.024** |
| E/E´-LV   1. day 7* 2. day 28 3. 36 weeks PMA | *60*  *34*  *36* | 6.6 (5.1/7.7)  7.4 (2.6)  7.5 (2.0) | 6.6 (5.4/9.8)  6.4 (1.8)  7.9 (2.5) | 6.6 (4.8/7.5)  7.6 (2.7)  7.3 (1.8) | 0.259  0.337  0.646 |
| **RV-PV Coupling** | | | | | |
| TAPSE/PAAT   1. day 7* 2. day 28 3. 36 weeks PMA | *41*  *32*  *30* | 0.08 (0.07/0.09)  0.1 (0.03)  0.16 (0.05) | 0.08 (0.07/0.13)  0.09 (0.03)  0.17 (0.04) | 0.08 (0.07/0.09)  0.10 (0.03)  0.15 (0.05) | 0.356  0.467  0.672 |
| **Pulmonary Artery Flow Characteristics** | | | | | |
| PAAT, ms   1. day 7 2. day 28 3. 36 weeks PMA* | *60*  *35*  *37* | 63.0 (20.1)  74.7 (18.1)  60 (52/72) | 50.2 (26.1)  67.8 (18.3)  56 (43/65) | 67.7 (15.1)  76.8 (17.9)  63 (52/67) | **0.002**  0.192  0.108 |
| RVET, ms   1. day 7 2. day 28 3. 36 weeks PMA* | *60*  *35*  *37* | 179 (21.9)  192 (13.6)  207 (193/216) | 175 (20.6)  188 (13.8)  206 (191/218) | 180 (22.4)  193 (13.5)  207 (193/216) | 0.332  0.384  0.917 |
| PAAT/RVET-Ratio   1. day 7 2. day 28 3. 36 weeks PMA | *60*  *35*  *37* | 0.36 (0.10)  0.39 (0.10)  0.31 (0.08) | 0.28 (0.13)  0.36 (0.10)  0.28 (0.08) | 0.38 (0.07)  0.40 (0.10)  0.32 (0.08) | **<0.001**  0.269  0.210 |

Data are demonstrated as absolute number with percentage, as absolute number with percentage or as mean values with standard deviation (SD, +/-) (normally distributed data) or median with IQR (25/75) (non-normally distributed data). Parameters with a p-level <0.05 are highlighted in bold. At T0 no echocardiographic assessments were included, as a standardized echocardiography could not be provided in many infants. At T1 echocardiographic assessments were performed in 100% of the infants, at T2 in 72%, and at T3 in 61% of the infants. Missing echocardiographic data occurred due to a) death prior to echocardiographic assessment, b) restlessness of the infant during echocardiographic assessment c) critical hemodynamic conditions and inability to perform a standardized echocardiography, d) death/ discharge of the infant prior to echocardiography. Abbreviations: GA: gestational age, LV: left ventricle, LVEF: left ventricular ejection fraction (Teichholtz approach), N: included samples, PAAT: pulmonary artery acceleration time, PDA: patent ductus arteriosus, PMA: post menstrual age, PV: pulmonary vasculature, RV: right ventricle, RVET: right ventricular ejection time, TAPSE: tricuspid annular plane systolic excursion). The asterisk is illustrating non-normally distributed data.
