# Supplementary figures and images for: Longitudinal evaluation of hemodynamic blood and echocardiographic biomarkers for the prediction of BPD and BPD-related pulmonary hypertension in very-low-birth-weight preterm infants
Source: Eur J Pediatr. 2024 Nov 15;184(1):15. doi: 10.1007/s00431-024-05841-8 (PMC11567987; doi:10.1007/s00431-024-05841-8)

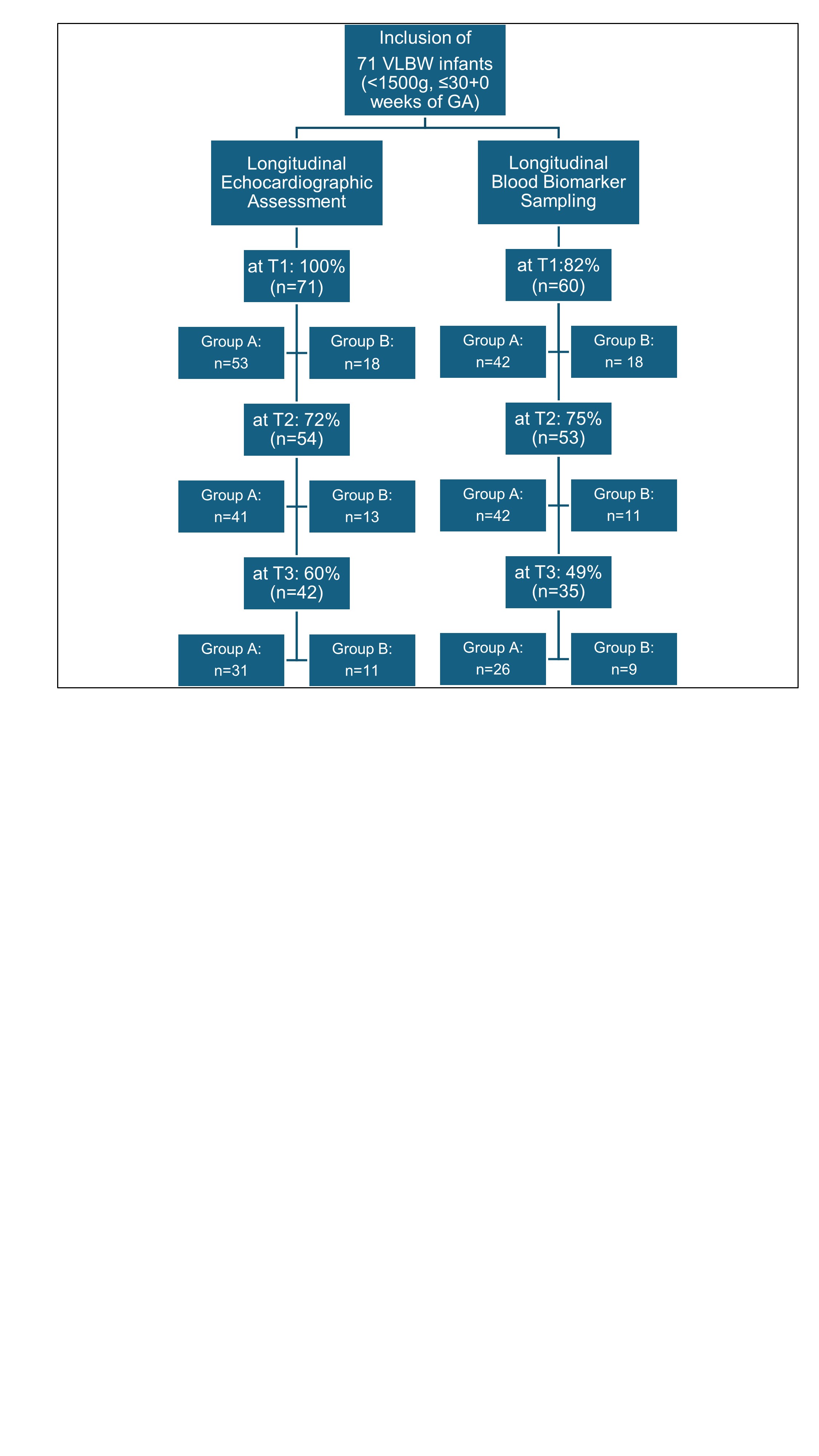

Supplement: Supplementary file 4 — Supplementary file4 (JPG 407 KB) [file 431_2024_5841_MOESM4_ESM.jpg]

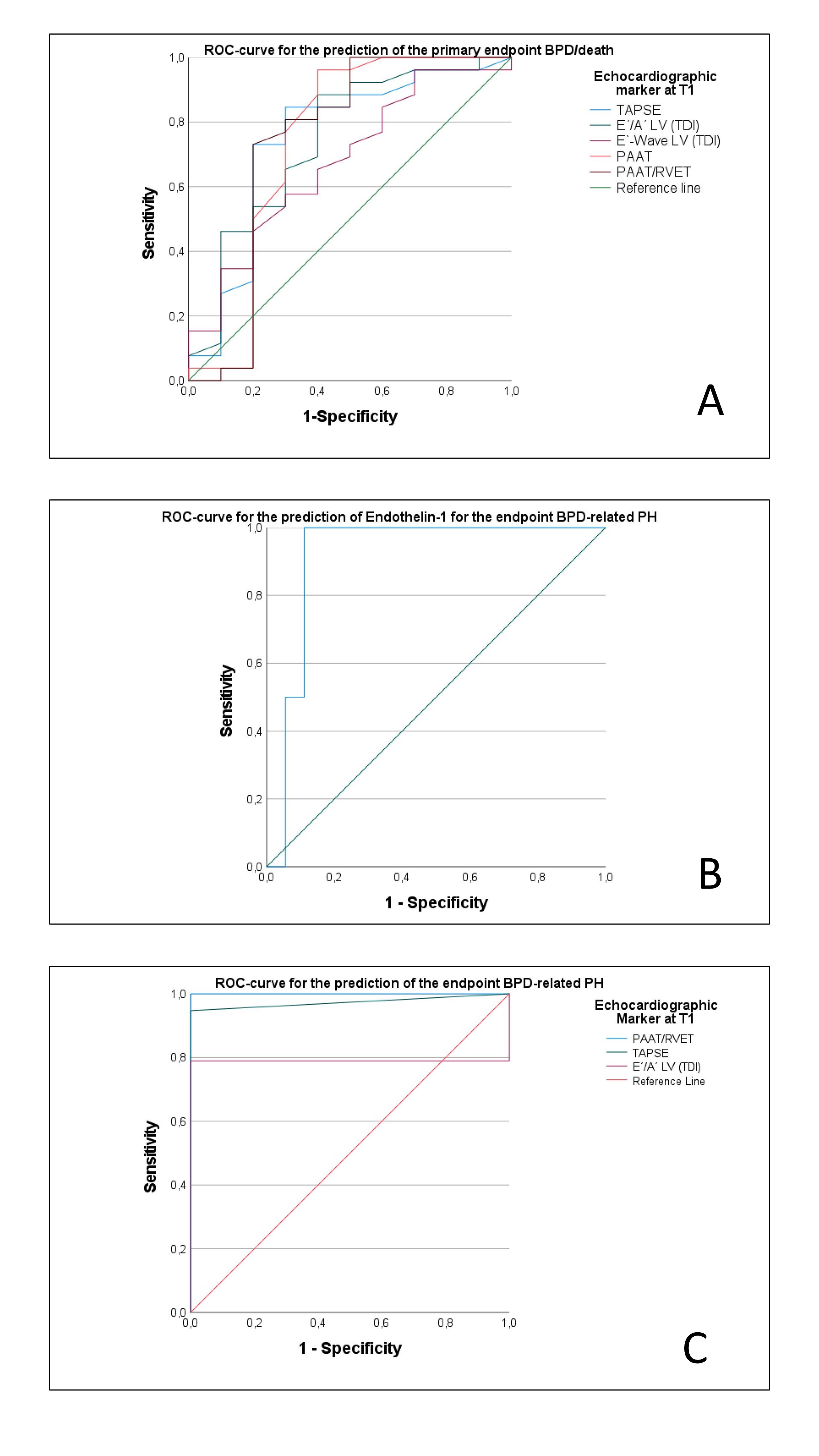

Supplement: Supplementary file 5 — Supplementary file5 (JPG 469 KB) [file 431_2024_5841_MOESM5_ESM.jpg]
